# Prognostic Benefit of New Drugs for HFrEF: A Systematic Review and Network Meta-Analysis

**DOI:** 10.3390/jcm11020348

**Published:** 2022-01-11

**Authors:** Matteo Pagnesi, Luca Baldetti, Alberto Aimo, Riccardo Maria Inciardi, Daniela Tomasoni, Enrico Vizzardi, Giuseppe Vergaro, Michele Emdin, Carlo Mario Lombardi

**Affiliations:** 1 Institute of Cardiology, ASST Spedali Civili di Brescia and Department of Medical and Surgical Specialties, Radiological Sciences and Public Health, University of Brescia, 25123 Brescia, Italy; m.pagnesi@gmail.com (M.P.); riccardo.inciardi@libero.it (R.M.I.); danielatomasoni8@gmail.com (D.T.); enrico.vizzardi@tin.it (E.V.); 2 Cardiac Intensive Care Unit, IRCCS San Raffaele Scientific Institute, 20132 Milan, Italy; luca.baldetti@gmail.com; 3 Institute of Life Sciences, Scuola Superiore Sant’Anna, 56127 Pisa, Italy; albertoaimo@libero.it (A.A.); vergaro@ftgm.it (G.V.); emdin@ftgm.it (M.E.); 4 Fondazione Toscana Gabriele Monasterio, 56124 Pisa, Italy

**Keywords:** heart failure, ejection fraction, network meta-analysis, SGLT2-inhibitors, vericiguat, omecamtiv mecarbil

## Abstract

Background: The new heart failure (HF) therapies of sodium-glucose cotransporter 2 inhibitors (SGLT2i), vericiguat, and omecamtiv mecarbil do not act primarily through the neuro-hormonal blockade, but have shown clinical benefits in patients with HF with reduced ejection fraction (HFrEF). However, their respective efficacies remain unclear. Our aim was to evaluate the relative efficacy of new drugs for HFrEF. Methods: We performed a network meta-analysis (NMA) of randomized controlled trials (RCTs) comparing SGLT2i, vericiguat, omecamtiv mecarbil, and placebo in HFrEF patients. The primary endpoint was the composite of cardiovascular death (CVD) or HF hospitalization (CVD-HF); secondary endpoints were CVD, all-cause death, and HF hospitalization (HFH). Results: Twelve RCTs (*n* = 23,861 patients) were included. A significant reduction in CVD-HF was observed with SGLT2i compared with placebo (risk ratio (RR) 0.77, 95% confidence interval (CI) 0.71–0.83), vericiguat (RR 0.84, 95% CI 0.75–0.93), and omecamtiv mecarbil (RR 0.80, 95% CI 0.72–0.88). No significant difference was observed between vericiguat and omecamtiv mecarbil (RR 0.95, 95% CI 0.87–1.04). SGLT2i were superior to placebo and omecamtiv mecarbil for all individual secondary endpoints (CVD, all-cause death, and HFH), and also to vericiguat for HFH. SGLT2i ranked as the most effective therapy for all endpoints, and vericiguat, omecamtiv mecarbil, and placebo ranked as the second, third, and last options, respectively, for the primary endpoint. Conclusions: In patients with HFrEF on standard-of-care therapy, SGLT2i therapy was associated with a reduced risk of CVD-HF compared to placebo, vericiguat, and omecamtiv mecarbil. Furthermore, SGLT2i were superior to placebo and omecamtiv mecarbil for CVD, all-cause death, and HFH, and also to vericiguat for HFH.

## 1. Introduction

Heart failure (HF) is a major cause of morbidity and mortality worldwide [1]. Medical therapies targeting the neuro-hormonal axes (classically represented by β-blockers, angiotensin-converting enzyme inhibitors (ACEi) or angiotensin receptor blockers (ARB), and mineralocorticoid receptor antagonists (MRA)) have significantly improved the clinical outcomes of patients with HF and reduced ejection fraction (HFrEF), and represent the mainstay of treatment for this condition [1,2,3]. The angiotensin receptor-neprilysin inhibitor (ARNI) sacubitril/valsartan has been proven to be superior to ACEi in HFrEF, and is recommended by HF guidelines, with American guidelines even recommending sacubitril/valsartan as the first-line therapy [2,3,4,5]. Over the last few years, further advances have been made in HFrEF pharmacotherapy with new drugs not acting directly through neuro-hormonal blockade (the sodium-glucose cotransporter 2 inhibitors (SGLT2i) dapagliflozin and empagliflozin, vericiguat, and omecamtiv mecarbil) showing a prognostic benefit in randomized controlled trials (RCTs) [6,7,8,9,10,11]. Of note, according to the latest European HF guidelines, SGLT2i are now considered as a first-line therapy for HFrEF, along with ACEi/ARNI, β-blockers, and MRA [3]. As head-to-head comparisons are lacking, and are unlikely to be performed in the future, the present network meta-analysis (NMA) aimed to evaluate the relative efficacy of SGLT2i, vericiguat, and omecamtiv mecarbil in patients with HFrEF.

## 2. Materials and Methods

Search strategy, study selection, and data extraction.

**Figure 1:**
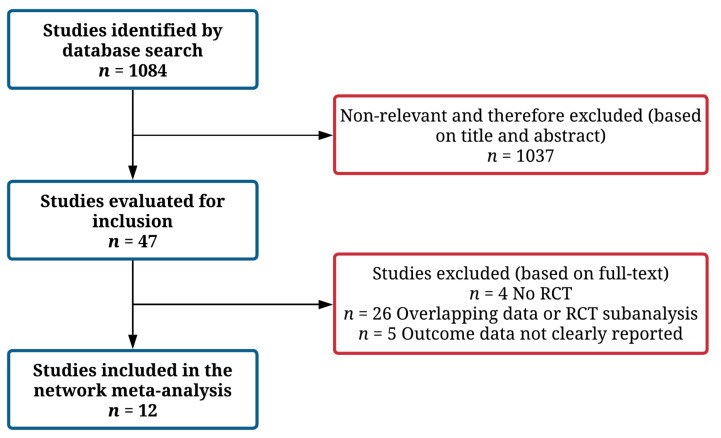
Study flow-chart.

Three authors (M.P., L.B. and D.T.) independently searched PubMed, Embase, Google Scholar, and the Cochrane Central Register of Controlled Trials (up to 18 March 2021), using the following combinations of keywords: “SGLT2” OR “dapagliflozin” OR “empagliflozin” OR “sotagliflozin” OR “vericiguat” OR “omecamtiv mecarbil” AND “heart failure”. Reference lists of the identified articles and pertinent reviews were also screened. All RCTs investigating SGLT2i, vericiguat, or omecamtiv mecarbil in patients with HFrEF were selected for inclusion. Studies including patients with acute decompensated HF or HF with preserved ejection fraction (as defined by investigators) were not included. Both phase 2 and phase 3 studies were considered for inclusion; furthermore, subgroup analyses from RCTs were also considered for inclusion. Studies with an observational design, not reporting data on primary or secondary endpoint at follow-up (as number of events and event rates), and reporting data on overlapping populations were excluded (Figure 1). Studies focused on sacubitril/valsartan were not considered for inclusion, as this drug was already included in 2016–2017 HF guidelines [2,5], targets the neuro-hormonal axis, and was already prescribed at baseline in a relevant proportion of patients enrolled in the other included trials (up to 40%).

The figure shows the study selection process. A total of 12 studies were included in the final analysis.

Two authors (M.P. and L.B.) independently assessed the identified studies for possible inclusion and performed data extraction (study designs, patient characteristics, and clinical outcomes). Conflicts regarding study inclusion, data extraction, and analysis were discussed and resolved with another author (C.M.L.). Two authors (D.T. and L.B.) assessed the risk of bias of the included studies using the Cochrane Collaboration tool (results available in Table S1).

This NMA was conducted according to Preferred Reporting Items for Systematic reviews and Meta-Analyses recommendations [12].

### 2.1. Study Endpoints

The primary endpoint was the composite of cardiovascular death (CVD) or HF hospitalization (CVD-HF). Secondary endpoints of interest were the following individual endpoints: CVD, all-cause death, and HF hospitalization (HFH).

### 2.2. Statistical Analysis

Treatment effects were compared with an NMA technique to provide more precise effect estimates, combining both direct and indirect evidence. In addition, this allowed for the comparison of pairs of interventions that were not directly assessed in randomized trials. This comprehensive comparison of all interventions in a single analysis also provided an estimation of their relative efficacy ranking for a given outcome [13,14,15]. This technique is extensively described in the Cochrane Handbook for Systematic Reviews of Interventions [15]. The present NMA included RCTs comparing the study drugs (SGLT2i, vericiguat, or omecamtiv mecarbil) with the placebo on top of standard-of-care therapy for HFrEF, thus obtaining indirect comparisons of the relative efficacy of the investigated study drugs [16,17]. The transitivity of the included studies was checked by a qualitative comparison of the baseline patient characteristics. A random-effects NMA was performed on the cumulative event rates for primary and secondary endpoints based on a frequentist approach with the DerSimonian−Laird estimator [18]. Effect estimates were based on relative risk (RR) per study, and were analyzed by considering their point estimates and 95% confidence interval (CI). The NMA results were summarized by means of league tables. No locally closed loop to calculate both the direct and indirect evidence exists to evaluate inconsistency.

To establish a relative ranking of the effectiveness of the available treatments, the surface under the cumulative ranking area (SUCRA) method and the probability of being the best treatment for a given outcome were calculated through a Bayesian approach [19]. Pre-specified sensitivity analyses were performed by including only phase 3 studies and by performing a random-effects NMA on hazard ratio (HR) estimates (instead of event counts).

The NMA was conducted in RStudio version 1.3.1093 (RStudio PBC, Boston, MA, USA) with the “netmeta” package for the frequentist approach and “bnma” package for the Bayesian analysis. Statistical significance was set at *p* value < 0.05 (two-sided) for the frequentist NMA.

## 3. Results

**Figure 2:**
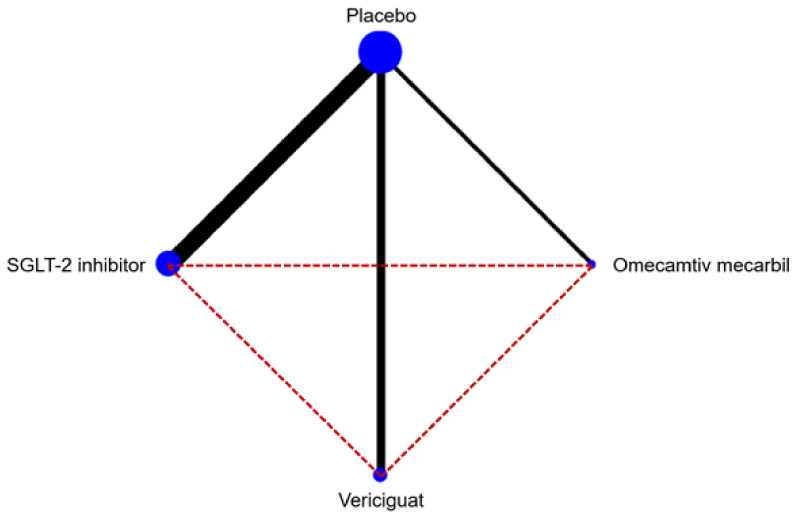
Network map of the study treatments.

As shown in Figure 1, the study selection process led to the final inclusion of 12 studies in the NMA, for an overall population of 23,861 patients [6,7,8,9,20,21,22,23,24,25,26,27]. The network map is available in Figure 2. The included trials compared SGLT2i (eight studies), vericiguat (two studies), and omecamtiv mecarbil (two studies) versus placebo, on top of standard medical therapy for HFrEF. As shown in Table 1, there were some differences regarding the study characteristics across the included trials (such as sample size, baseline NT-proBNP values, or percentage of patients already treated with ARNI).

### 3.1. Primary Endpoint

A total of seven studies (*n* = 22,694 patients) evaluated the primary endpoint of CVD-HF. Sample size, event counts, and summary measures are reported in Figure S1. Both SGLT2i and vericiguat were found to be superior to the placebo, while omecamtiv mecarbil was not (Figure S2). Furthermore, SGLT2i proved superior to vericiguat and omecamtiv mecarbil, whereas no significant difference was observed between vericiguat and omecamtiv mecarbil (Table 2).

In the probability analyses, SGLT2i had the highest probability of being the best agent to reduce CVD-HF, whereas vericiguat, omecamtiv mecarbil, and placebo ranked as the second, third, and worst therapies, respectively (Table 3 and Table S1).

### 3.2. Secondary Endpoints

A total of 10 studies (*n* = 23,550 patients) were available for the secondary endpoint of CVD (Figure S4). Only SGLT2i were proven to be superior to placebo, while vericiguat and omecamtiv mecarbil were not (Figure S5). SGLT2i were also superior to omecamtiv mecarbil, but not to vericiguat, and no significant difference was observed between vericiguat and omecamtiv mecarbil (Table 2). In the probability analyses, SGLT2i had the highest probability of being the best agent to reduce CVD (Table 3 and Figure S6).

A total of 12 studies (*n* = 23,861 patients) evaluated the secondary endpoint of all-cause death (Figure S7). Only SGLT2i were proven to be significantly more effective than placebo (Figure S8). SGLT2i were also proven to be superior to omecamtiv mecarbil, but not to vericiguat, and no significant difference was observed between vericiguat and omecamtiv mecarbil (Table 2). In the probability analyses, SGLT2i ranked as the best agent to reduce all-cause death (Table 3 and Figure S9).

A total of 10 studies (*n* = 23,445 patients) were available for the secondary endpoint of HFH (Figure S10). Only SGLT2i were found to be superior to the placebo (Figure S11). SGLT2i were also superior to vericiguat and omecamtiv mecarbil, whereas no difference was observed between vericiguat and omecamtiv mecarbil (Table 2). Again, SGLT2i had the highest probability of being the best agent to reduce HFH (Table 3 and Figure S12).

### 3.3. Sensitivity Analyses

A pre-specified random-effects NMA on HR estimates from the included studies was performed for the primary endpoint. A total of six studies were included. All three active treatments (SGLT2i, vericiguat, and omecamtiv mecarbil) were proven to be superior to the placebo (Figure S13), and SGLT2i were also superior to vericiguat and omecamtiv mecarbil (Table S2).

A pre-specified sensitivity analysis (random-effects NMA) including only phase 3 studies was also conducted for the primary endpoint. A total of four studies were included. Both SGLT2i and vericiguat were proven to be superior to the placebo (Figure S14). SGLT2i were also superior to vericiguat and omecamtiv mecarbil (Table S3).

## 4. Discussion

In our NMA including patients with HFrEF on standard medical therapy, SGLT2i (dapagliflozin/empagliflozin) were proven to be superior to the placebo, vericiguat, and omecamtiv mecarbil for the primary endpoint of CVD-HF. Furthermore, SGLT2i were proven to be superior to placebo and omecamtiv mecarbil for all secondary endpoints (CVD, all-cause death, and HFH), and also to vericiguat for the secondary endpoint of HFH. Accordingly, SGLT2i had the highest probability of being the best therapy to reduce all of the evaluated endpoints and ranked first in the probability analyses for all of the evaluated endpoints.

A variety of different drugs are becoming available in the treatment of HF, yet the relative superiorities over each other have not been formally investigated to date. In this NMA, we performed a quantitative assessment of drug efficacy on hard clinical endpoints in patients with HFrEF, on top of standard-of-care therapy based on ACEi/ARBs/ARNI, β-blockers, and MRA [2,3,5]. SGLT2i demonstrated a clear favorable effect in all of the investigated endpoints, a finding that further supports their role as potent disease-modifying drugs in HF and the recent proposal of an early start of SGLT2i therapy in HFrEF [28,29]. Indeed, SGLT2i were included as first-line therapy for HFrEF in the latest European HFrEF guidelines, along with neuro-hormonal antagonists (ACEi/ARNI, β-blockers, and MRA) [3]. Conversely, omecamtiv mecarbil and vericiguat are, at this time, intended for the treatment of patients with more advanced HFrEF.

In patients with type 2 diabetes mellitus, with or without a history of HF and cardiovascular disease, the use of SGLT2i (empagliflozin, dapagliflozin, and canagliflozin) has largely shown a reduction in the risk of HF hospitalization and an improvement in CV outcome [30]. The DApagliflozin and Prevention of Adverse Outcomes in Heart Failure (DAPA-HF) was the first randomized trial to investigate the benefits of dapagliflozin in a population with HFrEF, regardless of diabetes history. Dapagliflozin reduced the risk of CVD or worsening HF compared to the placebo (HR 0.74; 95% CI 0.65–0.85) [6]. More recently, the EMPagliflozin outcomE tRial in Patients With chrOnic heaRt Failure With Reduced Ejection Fraction (EMPEROR-Reduced) trial confirmed and expanded the positive results of DAPA-HF in patients with a more advanced disease (lower ejection fraction, higher natriuretic peptides levels, and worse renal function) [7]. In both trials, the benefits were primarily driven by a significant reduction in HF hospitalizations. A recent meta-analysis of these two large trials confirmed these promising results and demonstrated that treatment with SGLT2i led to a significant reduction in all-cause mortality (HR 0.87, 95% CI 0.77–0.98), CVD (HR 0.86, 95% CI 0.76–0.98), CVD-HF (HR 0.86, 95% CI 0.76–0.98), and renal outcome (HR 0.62, 95% CI 0.43–0.90) [31].

The mechanisms behind the beneficial effects of SGLT2i are not completely clear [32,33]. The levels of glycated haemoglobin, both at baseline and over time, do not seem to affect the course of treatment, suggesting favorable effects beyond glycemic control. SGLT2i also present diuretic properties—exerting their action on the proximal tubule, these drugs enhance glycosuria and natriuresis and ensure osmotic diuresis, which is more pronounced in diabetic patients [32,34]. The hemodynamic consequence with a reduction in preload and decongestion might justify the prominent reduction in HF hospitalizations. However, SGLT2i could also improve cardiomyocyte metabolism and blunt the progression of myocardial fibrosis, leading to an improved diastolic function and reverse cardiac remodeling [32,35]. The recent Effect of Empagliflozin on Left Ventricular Volumes in Patients with Type 2 Diabetes, or Prediabetes, and Heart Failure with Reduced Ejection Fraction (SUGAR-DM-HF) trial showed that empagliflozin therapy caused a significant reduction in left ventricular volumes compared to the placebo, even if without an improvement in global longitudinal strain, after 36 weeks of treatment [26]. Similar results were observed after 12 weeks of treatment in a sub-study of the Empagliflozin in Heart Failure Patients with Reduced Ejection Fraction (Empire HF) trial [36]. Furthermore, a rapid reduction in pulmonary artery pressures was recently demonstrated with empagliflozin in patients with HF and CardioMEMS pulmonary artery pressure sensor, independently of diuretic management [37]. SGLT2i are generally safe and well tolerated, with genital tract infections being the most common adverse event, while hypotension, hyperkalaemia, and renal dysfunction, the most feared adverse effects of neuro-hormonal antagonists, have a similar incidence in patients treated with SGLTi or placebo [6,7].

In our NMA, besides the superiority of SGLT2i over placebo in HFrEF, we found a significant reduction in the primary endpoint of CVD-HF with SGLT2i compared to vericiguat and omecamtiv mecarbil, two drugs that were recently associated with benefits compared to the placebo in the Vericiguat Global Study in Subjects with Heart Failure with Reduced Ejection Fraction (VICTORIA) and Global Approach to Lowering Adverse Cardiac Outcomes through Improving Contractility in Heart Failure (GALACTIC-HF) trials, respectively [8,9]. The mechanism associated with the benefits of vericiguat in HFrEF is a direct stimulation of the soluble guanylate cyclase, sensitizing it to endogenous nitric oxide and leading to an enhancement of the cyclic guanosine monophosphate pathway, with positive effects on hemodynamics and vascular and myocardial function [8,23]. Conversely, omecamtiv mecarbil is a cardiac myosin activator that ameliorates myocardial function and contractility by direct improvement of the cardiac sarcomere function [9,22]. It is important to underline that this superiority of SGLT2i over vericiguat and omecamtiv mecarbil was based only on indirect comparisons. Furthermore, some heterogeneity in the baseline characteristics of the included RCTs may be responsible for some of the observed differences: for example, left ventricular ejection fraction and use of ARNI at baseline tended to be slightly higher in SGLT2i trials, whereas median NT-proBNP values were higher and patients were less stable in vericiguat trials.

Recent NMA studies have focused on omecamtiv mecarbil and tested this drug in the comparisons. Of note, we found a superiority of SGLT2i over placebo, vericiguat, and omecamtiv mecarbil for CVD-HF, hence supporting the use of SGLT2i in HFrEF patients already treated with conventional neuro-hormonal blockers.

### Limitations

A relevant limitation of the present analysis is that all comparisons between SGLT2i, omecamtiv mecarbil, and vericiguat are indirect, as trials directly comparing these treatments have not been performed to date (and are unlikely to be performed in the future). Nonetheless, NMA is an established tool to indirectly compare the relative efficacy of different therapies in the absence of RCTs involving direct comparisons between them [38]. Furthermore, although most patients were randomized upon optimized medical therapy, some differences in the baseline characteristics and medical treatments across trials may have contributed to the observed superiority among different drugs. For example, the different rate of ARNI prescription across the included studies could be particularly relevant, as ARNI is already part of the standard-of-care therapy for HFrEF [2,5], and the prognostic impact of novel drugs should be tested on a similar background of baseline medical therapy for HF. Furthermore, the SGLT2i trials included only 25–30% of patients with NYHA class III−IV [6,7], whereas the omecamtiv mecarbil and vericiguat trials included up to 45% of patients with NYHA III−IV [8,9]. Another potential limitation may be related to differences between empagliflozin and dapagliflozin, leading to non-class effects of SGLT2i, an issue that is not addressed by our analysis.

## 5. Conclusions

SGLT2i were associated with a reduced risk of CVD-HF compared to placebo, vericiguat, and omecamtiv mecarbil, given on top of standard therapy for HFrEF. Furthermore, SGLT2i were superior to placebo and omecamtiv mecarbil for CVD, all-cause death, and HFH, and also to vericiguat for HFH.

## Figures and Tables

**Table 1:** Main characteristics of the included studies.

Study	Year	Treatment	*n* Patients	Age (Years)	Male Sex (%)	EF (%)	Diabetes (%)	NT-proBNP (pg/mL)	Background HF Therapy				Follow-Up
									ACEi/ARB (%)	Beta-Blocker (%)	ARNI (%)	MRA (%)	
GALACTIC-HF [9]	2021	Omecamtiv mecarbil vs. Placebo	8232	65	79	27	40	1971	87 *	94	19	78	22 months (median)
COSMIC-HF [22]	2016	Omecamtiv mecarbil vs. Placebo	298	63	82	29	39	1719	93	97	0	61	24 weeks
VICTORIA [8]	2020	Vericiguat vs. Placebo	5050	67	76	29	47	2816	73	93	15	70	11 months (median)
SOCRATES-REDUCED [23]	2015	Vericiguat vs. Placebo	183	68	82	29	49	3076	81	92	0	62	12 weeks
EMPEROR-Reduced [7]	2020	Empagliflozin vs. Placebo	3730	67	76	27	50	1907	70	95	19	71	16 months (median)
EMPERIAL-Reduced [24]	2020	Empagliflozin vs. Placebo	311	70	74	30	60	1489	55	95	37	58	12 weeks
Empire HF [25]	2020	Empagliflozin vs. Placebo	190	64	85	30	17	594	96 *	95	31	66	12 weeks
SUGAR-DM-HF [26]	2021	Empagliflozin vs. Placebo	105	69	73	33	78	466	61	91	34	60	40 weeks
EMPA-TROPISM (ATRU-4) [27]	2021	Empagliflozin vs. Placebo	84	62	64	36	0	NA	42	88	43	33	6 months
DAPA-HF [6]	2019	Dapagliflozin vs. Placebo	4744	66	77	31	42	1437	84	96	11	71	18 months (median)
DECLARE-TIMI 58 (HFrEF subgroup) [20]	2019	Dapagliflozin vs. Placebo	671	63	84	38	100	NA	88	88	NA	30	4.2 years (median)
DEFINE-HF [21]	2019	Dapagliflozin vs. Placebo	263	61	73	26	62	1136	59	97	33	61	12 weeks

* ACEi, ARB, or ARNI. ACEi—angiotensin-converting enzyme inhibitors; ARB—angiotensin receptor blockers; ARNI—angiotensin receptor-neprilysin inhibitor; EF—ejection fraction; HF—heart failure; MRA—mineralocorticoid receptor antagonist; NA—not available; NT-proBNP—N-terminal pro-B-type natriuretic peptide. This graph shows available comparisons between study treatments (with respect to the primary endpoint). The bullet diameter represents the size of the included randomized controlled trials, and line thickness represents the number of trials with direct comparisons. Direct comparisons are represented by continuous lines, while indirect comparisons are represented by dashed lines.

**Table 2:** League table showing pooled risk ratios for primary and secondary endpoints.

Endpoint	Placebo	SGLT2i	Vericiguat	Omecamtiv Mecarbil
CV death or HF hospitalization				
	Placebo	0.77 (0.71–0.83)	0.92 (0.85–0.99)	0.96 (0.91–1.02)
	1.30 (1.20–1.41)	SGLT2i	1.19 (1.07–1.33)	1.25 (1.13–1.39)
	1.09 (1.01–1.17)	0.84 (0.75–0.93)	Vericiguat	1.05 (0.96–1.15)
	1.04 (0.98–1.10)	0.80 (0.72–0.88)	0.95 (0.87–1.04)	Omecamtiv mecarbil
CV death				
	Placebo	0.85 (0.75–0.96)	0.94 (0.83–1.06)	1.01 (0.93–1.10)
	1.18 (1.04–1.33)	SGLT2i	1.10 (0.93–1.31)	1.19 (1.03–1.38)
	1.07 (0.95–1.21)	0.91 (0.76–1.08)	Vericiguat	1.08 (0.93–1.25)
	0.99 (0.91–1.08)	0.84 (0.72–0.98)	0.93 (0.80–1.08)	Omecamtiv mecarbil
All-cause death				
	Placebo	0.86 (0.77–0.95)	0.96 (0.86–1.07)	1.00 (0.93–1.07)
	1.16 (1.05–1.29)	SGLT2i	1.11 (0.96–1.29)	1.16 (1.02–1.32)
	1.05 (0.94–1.16)	0.90 (0.77–1.04)	Vericiguat	1.04 (0.92–1.19)
	1.00 (0.93–1.08)	0.86 (0.76–0.98)	0.96 (0.84–1.09)	Omecamtiv mecarbil
HF hospitalization				
	Placebo	0.73 (0.66–0.81)	0.92 (0.84–1.00)	0.97 (0.90–1.04)
	1.37 (1.24–1.52)	SGLT2i	1.26 (1.10–1.44)	1.33 (1.17–1.50)
	1.09 (1.00–1.19)	0.79 (0.69–0.91)	Vericiguat	1.05 (0.94–1.18)
	1.03 (0.97–1.11)	0.75 (0.67–0.85)	0.95 (0.85–1.06)	Omecamtiv mecarbil

Values are reported as pooled risk ratios and 95% confidence intervals. The pooled effect estimates obtained from the network meta-analysis are reported for column intervention relative to raw. CV—cardiovascular; HF—heart failure; SGLT2i—sodium-glucose cotransporter 2 inhibitors.

**Table 3:** Probability ranks for primary and secondary endpoints.

Treatment	P_best_	SUCRA
CV death or HF hospitalization		
Placebo	0.29	3.91
SGLT2i	77.24	99.97
Vericiguat	15.92	61.54
Omecamtiv mecarbil	6.55	34.58
CV death		
Placebo	1.49	24.76
SGLT2i	61.14	95.09
Vericiguat	25.89	60.85
Omecamtiv mecarbil	11.48	19.30
Any death		
Placebo	3.66	23.49
SGLT2i	64.97	96.92
Vericiguat	28.40	53.75
Omecamtiv mecarbil	2.97	25.83
HF hospitalization		
Placebo	0.48	6.40
SGLT2i	78.21	99.99
Vericiguat	19.12	59.60
Omecamtiv mecarbil	2.19	34.01

CV—cardiovascular; HF—heart failure; P_best_—probability of each treatment being the best (%); SGLT2i—sodium-glucose cotransporter 2 inhibitors; SUCRA—surface under the cumulative ranking.

## Data Availability

Data supporting the study results can be derived from the original publications included in our network meta-analysis.

## Supplementary Materials

The following are available online at https://www.mdpi.com/article/10.3390/jcm11020348/s1, Figure S1: Forest plot summarizing data from individual studies for primary endpoint (main analysis), Figure S2: Forest plot of each treatment versus PLACEBO for primary endpoint (main analysis), Figure S3: Cumulative probability rank plots for each treatment being the best with respect to primary endpoint (main analysis), Figure S4: Forest plot summarizing data from individual studies for CV death (main analysis), Figure S5: Forest plot of each treatment versus PLACEBO for CV death (main analysis), Figure S6: Cumulative probability rank plots for each treatment being the best with respect to CV death (main analysis), Figure S7: Forest plot summarizing data from individual studies for all-cause death (main analysis), Figure S8: Forest plot of each treatment versus PLACEBO for all-cause death (main analysis), Figure S9: Cumulative probability rank plots for each treatment being the best with respect to all-cause death (main analysis), Figure S10: Forest plot summarizing data from individual studies for HF hospitalization (main analysis), Figure S11: Forest plot of each treatment versus PLACEBO for HF hospitalization (main analysis), Figure S12: Cumulative probability rank plots for each treatment being the best with respect to HF hospitalization (main analysis), Figure S13: Forest plot of each treatment versus PLACEBO for primary endpoint (sensitivity analysis – NMA on HR estimates), Table S1: Risk of bias of individual studies by revised Cochrane Risk Assessment tool, Table S2: League table showing pooled HRs for primary and secondary endpoints (sensitivity analysis – NMA on HR estimates), Table S3: League table showing pooled risk ratios for primary and secondary endpoints (sensitivity analysis – NMA including phase 3 trials only).

## Abbreviations

ACEi	angiotensin-converting enzyme inhibitor
ARB	angiotensin receptor blocker
ARNI	angiotensin receptor-neprilysin inhibitor
CI	confidence interval
CVD	cardiovascular death
CVD-HF	cardiovascular death or heart failure hospitalization
HF	heart failure
HFH	heart failure hospitalization
HR	hazard ratio
HFrEF	heart failure with reduced ejection fraction
MRA	mineralocorticoid receptor antagonist
