# Supplementary material for: Prognostic Benefit of New Drugs for HFrEF: A Systematic Review and Network Meta-Analysis
Source: J Clin Med. 2022 Jan 11;11(2):348. doi: 10.3390/jcm11020348 (PMC8777808; doi:10.3390/jcm11020348)

**SUPPLEMENTARY**

**Supplementary Table S1 – Risk of bias of individual studies by revised Cochrane Risk Assessment tool**

| Study name | Bias arising from the randomization process | Bias due to deviations from intended interventions | Bias due to missing outcome data | Bias in measurement of the outcome | Bias in selection of the reported result |
|---|---|---|---|---|---|
| GALACTIC-HF | - | - | - | - | - |
| COSMIC-HF | - | - | - | - | - |
| VICTORIA | - | - | - | - | - |
| SOCRATES-REDUCEI | - | - | - | - | - |
| EMPEROR-Reduced | - | - | - | - | - |
| EMPERIAL-Reduced | - | - | - | - | - |
| Empire HF | - | - | - | - | +/- |
| SUGAR-DM-HF | - | - | - | - | +/- |
| EMPA-TROPISM (ATRU-4) | - | - | - | - | +/- |
| DAPA-HF | - | - | - | - | - |
| DECLARE-TIMI 58 subgroup HFrEF | - | - | - | - | - |
| DEFINE-HF | - | - | - | - | - |
| TOTAL | - | - | - | - | - |

Each field should be graded as: Low - /High + /Some concerns +/- (please use the corresponding signs pasting them: -/+/-/ +)

**Information at:**

https://methods.cochrane.org/bias/resources/cochrane-risk-bias-tool

https://sites.google.com/site/riskofbiastool/welcome/rob-2-0-tool?authuser=0

https://sites.google.com/site/riskofbiastool/welcome/rob-2-0-tool/current-version-of-rob-2?authuser=0

**Supplementary Table S2: League table showing pooled HRs for primary and secondary endpoints (sensitivity analysis – NMA on HR estimates).**

| Endpoint | Placebo | SGLT2i | Vericiguat | Omecamtiv mecarbil |
|---|---|---|---|---|
| CV death or HF hospitalization | | | | |
| | Placebo | 0.74 (0.67-0.81) | 0.89 (0.82-0.98) | 0.92 (0.86-0.99) |
| | 1.35 (1.23-1.49) | SGLT2i | 1.21 (1.06-1.38) | 1.25 (1.11-1.40) |
| | 1.12 (1.02-1.23) | 0.83 (0.73-0.94) | Vericiguat | 1.03 (0.92-1.16) |
| | 1.09 (1.01-1.17) | 0.80 (0.71-0.90) | 0.97 (0.86-1.09) | Omecamtiv mecarbil |
| CV death | | | | |
| | Placebo | 0.82 (0.67-0.99) | 0.93 (0.71-1.21) | 1.01 (0.79-1.29) |
| | 1.23 (1.01-1.49) | SGLT2i | 1.14 (0.82-1.58) | 1.24 (0.90-1.69) |
| | 1.08-0.83-1.40) | 0.88 (0.63-1.22) | Vericiguat | 1.09 (0.76-1.56) |
| | 0.99 (0.77-1.27) | 0.81 (0.59-1.11) | 0.92 (0.64-1.32) | Omecamtiv mecarbil |
| All-cause death | | | | |
| | Placebo | 0.82 (0.69-0.98) | 0.95 (0.74-1.22) | 1.00 (0.79-1.26) |
| | 1.22 (1.02-1.46) | SGLT2i | 1.16 (0.85-1.57) | 1.22 (0.91-1.64) |
| | 1.05 (0.82-1.35) | 0.86 (0.64-1.17) | Vericiguat | 1.05 (0.75-1.48) |
| | 1.00 (0.79-1.26) | 0.82 (0.61-1.10) | 0.95 (0.68-1.34) | Omecamtiv mecarbil |
| HF hospitalization | | | | |
| | Placebo | 0.69 (0.62-0.77) | 0.90 (0.81-1.00) | 0.95 (0.87-1.03) |
| | 1.45 (1.30-1.62) | SGLT2i | 1.30 (1.12-1.52) | 1.38 (1.20-1.58) |
| | 1.11 (1.00-1.23) | 0.77 (0.66-0.89) | Vericiguat | 1.06 (0.92-1.21) |
| | 1.05 (0.97-1.15) | 0.73 (0.63-0.84) | 0.95 (0.83-1.08) | Omecamtiv mecarbil |

Values are reported as pooled hazard ratios and 95% confidence intervals. The pooled effect estimates obtained from the network meta-analysis are reported for column intervention relative to raw.
HR = hazard ratio; NMA = network meta-analysis.

**Supplementary Table S3: League table showing pooled risk ratios for primary and secondary endpoints (sensitivity analysis – NMA including phase 3 trials only).**

| Endpoint | Placebo | SGLT2i | Vericiguat | Omecamtiv mecarbil |
|---|---|---|---|---|
| CV death or HF hospitalization | | | | |
| | Placebo | 0.78 (0.71-0.85) | 0.92 (0.86-0.99) | 0.96 (0.91-1.02) |
| | 1.29 (1.18-1.40) | SGLT2i | 1.19 (1.06-1.33) | 1.24 (1.12-1.38) |
| | 1.08 (1.01-1.17) | 0.84 (0.75-0.94) | Vericiguat | 1.05 (0.95-1.15) |
| | 1.04 (0.98-1.09) | 0.81 (0.73-0.89) | 0.96 (0.87-1.05) | Omecamtiv mecarbil |
| CV death | | | | |
| | Placebo | 0.87 (0.77-0.99) | 0.94 (0.83-1.06) | 1.01 (0.93-1.10) |
| | 1.15 (1.01-1.30) | SGLT2i | 1.08 (0.90-1.28) | 1.16 (0.99-1.35) |
| | 1.07 (0.94-1.02) | 0.93 (0.78-1.11) | Vericiguat | 1.08 (0.93-1.25) |
| | 0.99 (0.91-1.08) | 0.86 (0.74-1.01) | 0.93 (0.80-1.08) | Omecamtiv mecarbil |
| All-cause death | | | | |
| | Placebo | 0.88 (0.79-0.99) | 0.96 (0.86-1.07) | 1.00 (0.99-1.08) |
| | 1.13 (1.01-1.26) | SGLT2i | 1.08 (0.93-1.27) | 1.13 (0.99-1.29) |
| | 1.04 (0.94-1.16) | 0.92 (0.79-1.08) | Vericiguat | 1.04 (0.91-1.19) |
| | 1.00 (0.93-1.08) | 0.88 (0.77-1.01) | 0.96 (0.84-1.09) | Omecamtiv mecarbil |
| HF hospitalization | | | | |
| | Placebo | 0.72 (0.65-0.81) | 0.92 (0.85-1.01) | 0.97 (0.90-1.04) |
| | 1.38 (1.24-1.54) | SGLT2i | 1.28 (1.11-1.47) | 1.34 (1.17-1.52) |
| | 1.08 (0.99-1.18) | 0.78 (0.68-0.90) | Vericiguat | 1.05 (0.94-1.17) |
| | 1.03 (0.97-1.11) | 0.75 (0.66-0.85) | 0.96 (0.86-1.07) | Omecamtiv mecarbil |

Values are reported as pooled risk ratios and 95% confidence intervals. The pooled effect estimates obtained from the network meta-analysis are reported for column intervention relative to raw.

**Supplementary Figure S1: Forest plot summarizing data from individual studies for primary endpoint (main analysis).**

| Study | Experimental Events | Experimental Total | Control Events | Control Total | RR | 95%−CI |
|---|---|---|---|---|---|---|
| EMPA−TROPISM (ATRU−4) | 0 | 42 | 3 | 42 | 0.03 | [0.00; 17.26] |
| SOCRATES−REDUCED | 10 | 91 | 18 | 92 | 0.56 | [0.27; 1.15] |
| DECLARE−TIMI 58 subgroup HFrEF | 59 | 318 | 95 | 353 | 0.69 | [0.52; 0.92] |
| DAPA−HF | 382 | 2373 | 495 | 2371 | 0.77 | [0.68; 0.87] |
| EMPEROR−Reduced | 361 | 1863 | 462 | 1867 | 0.78 | [0.69; 0.88] |
| VICTORIA | 897 | 2526 | 972 | 2524 | 0.92 | [0.86; 0.99] |
| GALACTIC−HF | 1453 | 4120 | 1504 | 4112 | 0.96 | [0.91; 1.02] |

Heterogeneity: $I^2 = 74\%$ [45%; 88%], $p < 0.01$

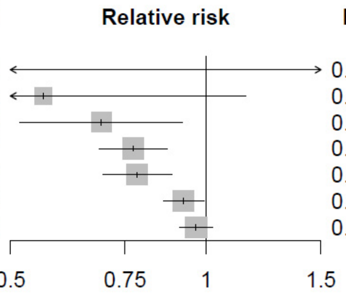

**Supplementary Figure S2: Forest plot of each treatment versus PLACEBO for primary endpoint (main analysis).**

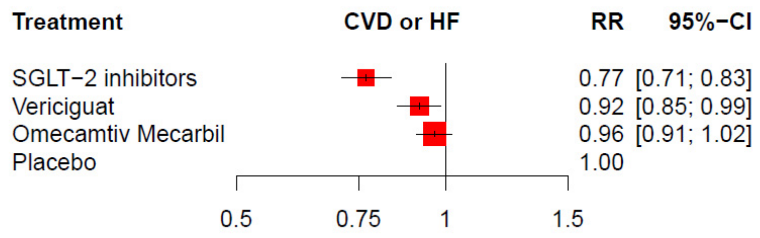

**Supplementary Figure S3: Cumulative probability rank plots for each treatment being the best with respect to primary endpoint (main analysis).**

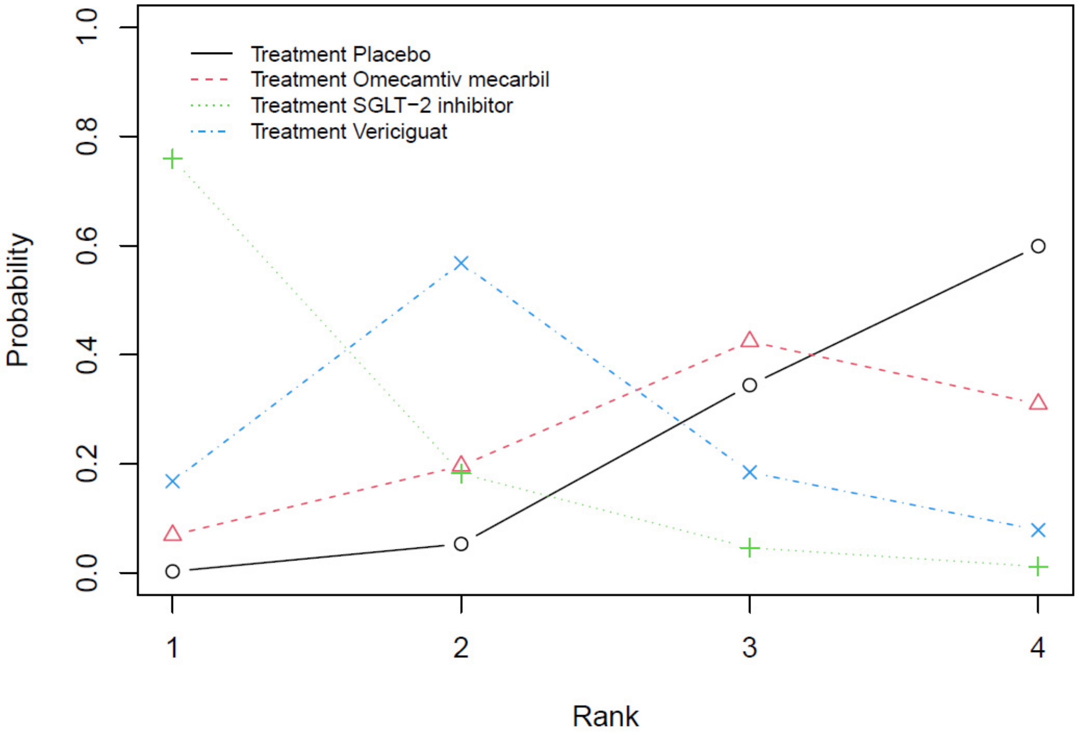

**Supplementary Figure S4: Forest plot summarizing data from individual studies for CV death (main analysis).**

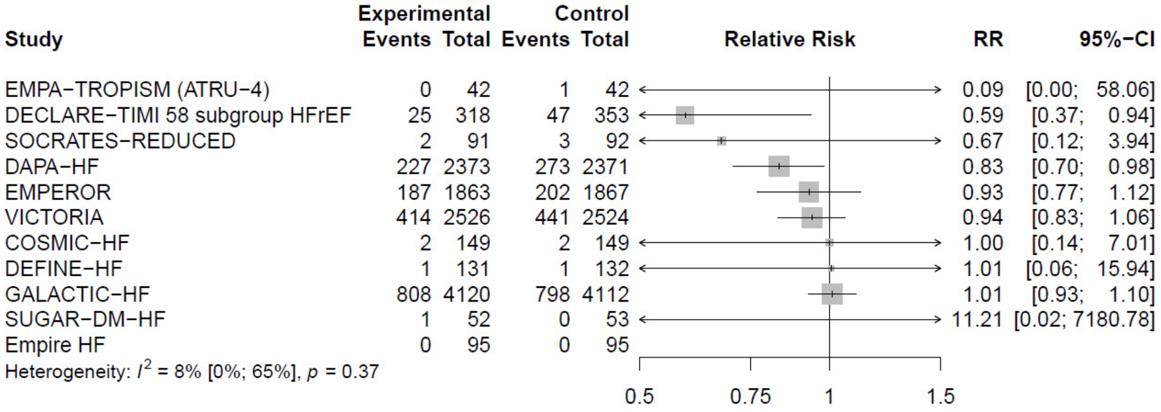

**Supplementary Figure S5: Forest plot of each treatment versus PLACEBO for CV death (main analysis).**

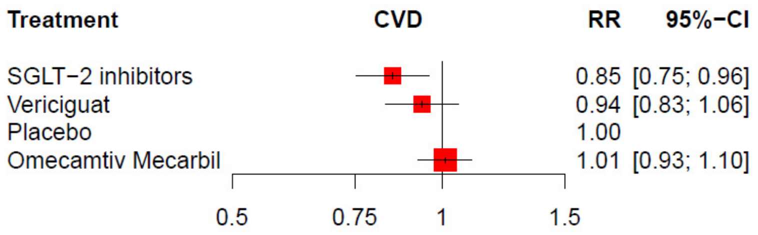

**Supplementary Figure S6: Cumulative probability rank plots for each treatment being the best with respect to CV death (main analysis).**

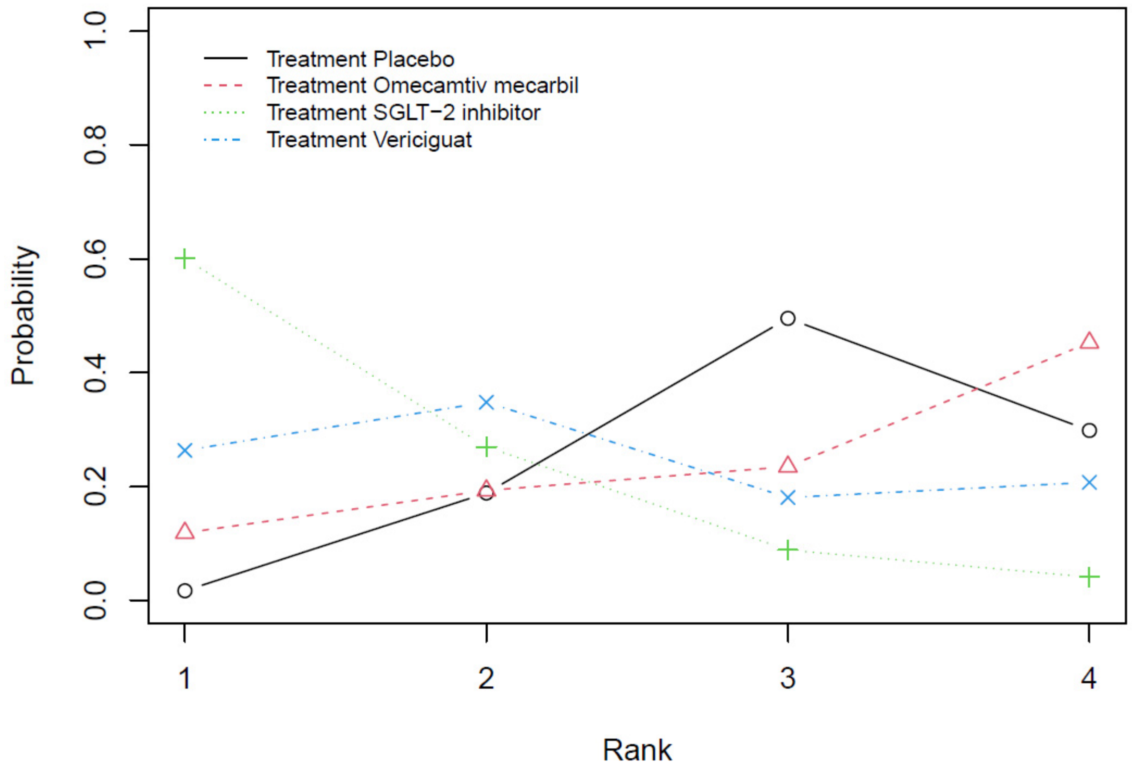

**Supplementary Figure S7: Forest plot summarizing data from individual studies for all-cause death (main analysis).**

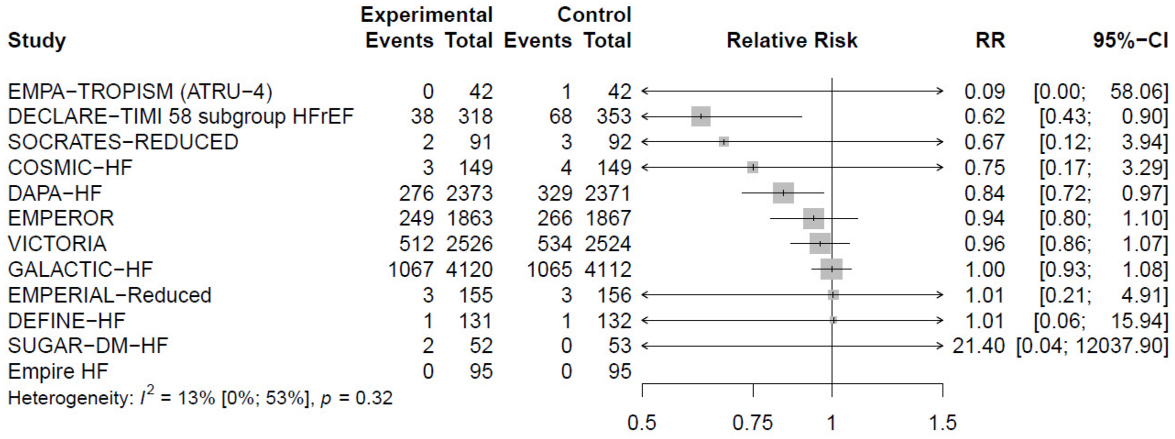

**Supplementary Figure S8: Forest plot of each treatment versus PLACEBO for all-cause death (main analysis).**

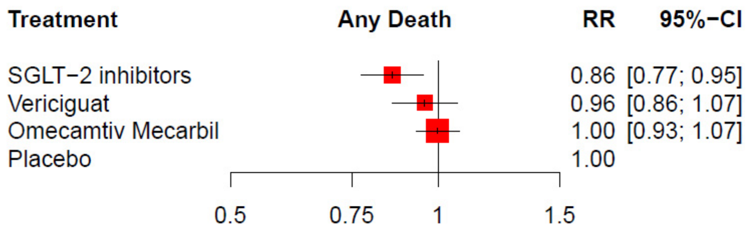

**Supplementary Figure S9: Cumulative probability rank plots for each treatment being the best with respect to all-cause death (main analysis).**

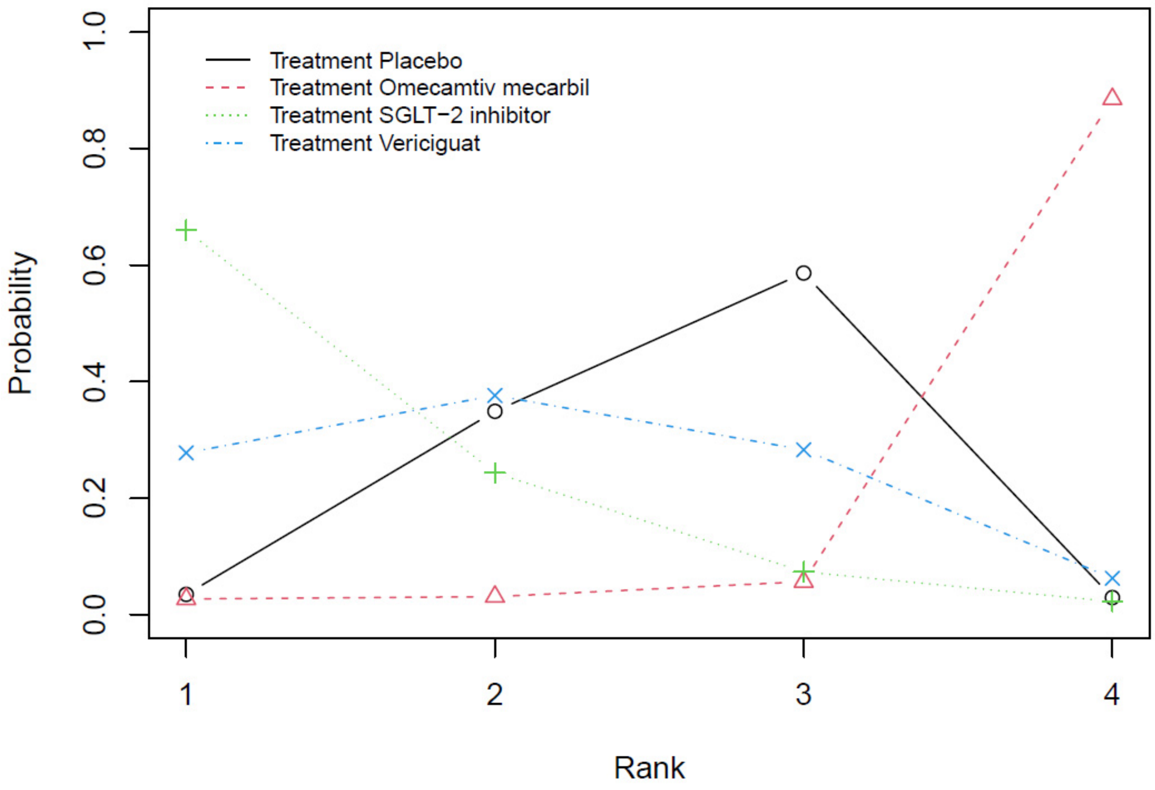

**Supplementary Figure S10: Forest plot summarizing data from individual studies for HF hospitalization (main analysis).**

| Study | Experimental Events | Experimental Total | Control Events | Control Total | RR | 95%-CI |
|---|---|---|---|---|---|---|
| EMPA−TROPISM (ATRU−4) | 0 | 42 | 2 | 42 | 0.05 | [0.00; 26.71] |
| SOCRATES−REDUCED | 9 | 91 | 16 | 92 | 0.57 | [0.27; 1.22] |
| EMPEROR | 246 | 1863 | 342 | 1867 | 0.72 | [0.62; 0.84] |
| DECLARE−TIMI 58 subgroup HFrEF | 41 | 318 | 63 | 353 | 0.72 | [0.50; 1.04] |
| DAPA−HF | 231 | 2373 | 318 | 2371 | 0.73 | [0.62; 0.85] |
| COSMIC−HF | 10 | 149 | 11 | 149 | 0.91 | [0.40; 2.08] |
| VICTORIA | 691 | 2526 | 747 | 2524 | 0.92 | [0.85; 1.01] |
| GALACTIC−HF | 1142 | 4120 | 1179 | 4112 | 0.97 | [0.90; 1.04] |
| DEFINE−HF | 10 | 131 | 8 | 132 | 1.26 | [0.51; 3.09] |
| Empire HF | 1 | 95 | 0 | 95 | 11.00 | [0.02; 7081.21] |

Heterogeneity: $I^2$ = 63% [27%; 81%], $p < 0.01$

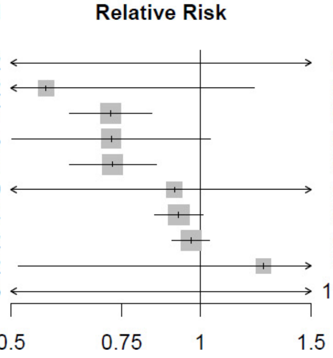

**Supplementary Figure S11: Forest plot of each treatment versus PLACEBO for HF hospitalization (main analysis).**

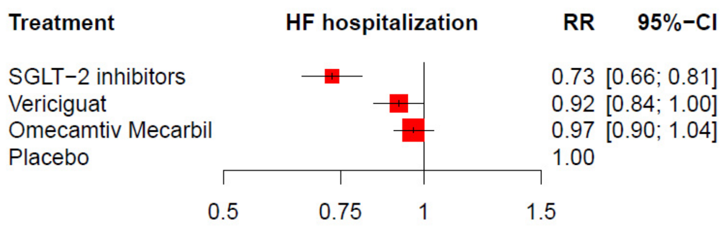

**Supplementary Figure S12: Cumulative probability rank plots for each treatment being the best with respect to HF hospitalization (main analysis).**

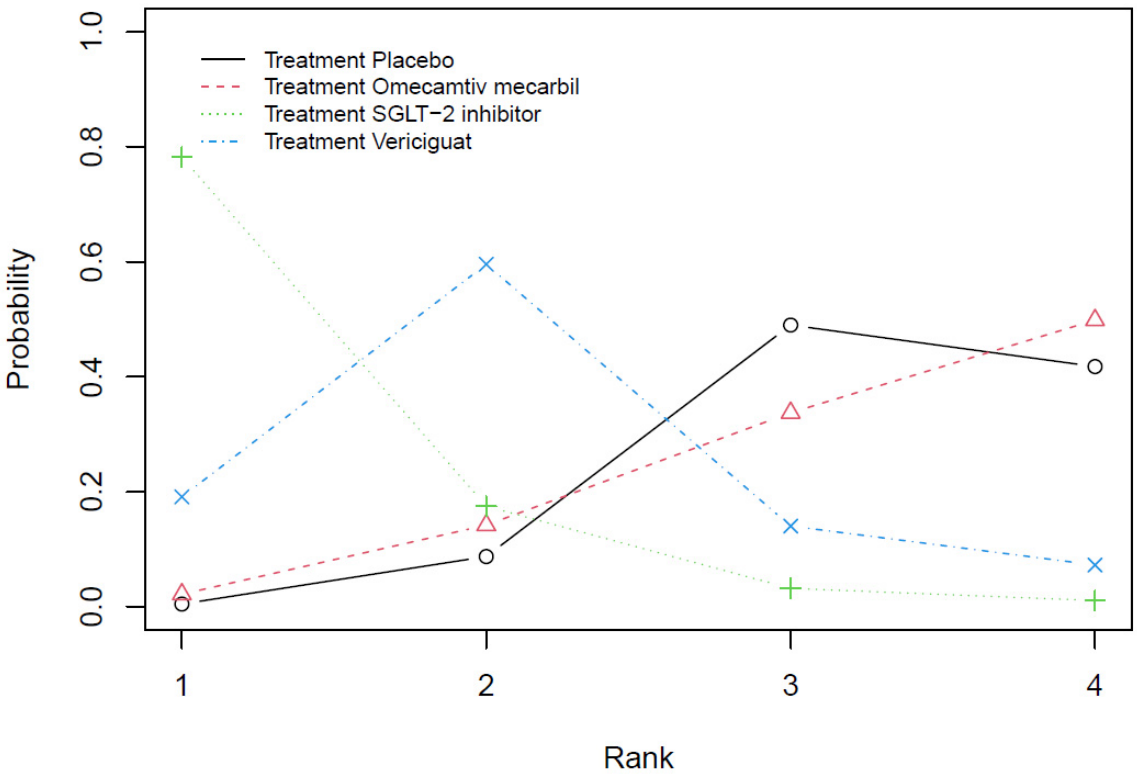

**Supplementary Figure S13: Forest plot of each treatment versus PLACEBO for primary endpoint (sensitivity analysis – NMA on HR estimates).**

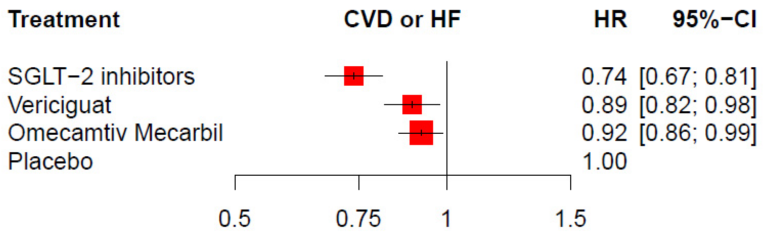

**Supplementary Figure S14: Forest plot of each treatment versus PLACEBO for primary endpoint (sensitivity analysis – NMA including phase 3 trials only).**

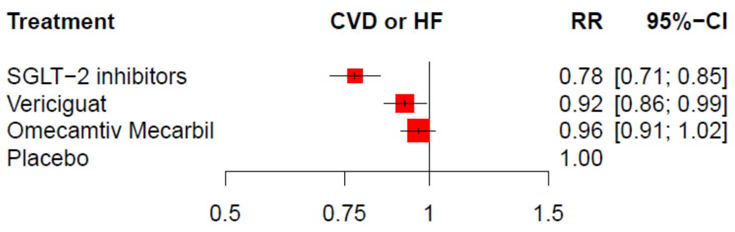

Supplement: Supplementary file 1 [file jcm-11-00348-s001.zip › jcm-1482370-supplementary.pdf]
